# Meta-Analysis of the Relationship between XRCC1-Arg399Gln and Arg280His Polymorphisms and the Risk of Prostate Cancer

**DOI:** 10.1038/srep09905

**Published:** 2015-04-30

**Authors:** Jie Yan, Xiantao Wang, Hui Tao, Zengfu Deng, Wang Yang, Faquan Lin

**Affiliations:** 1 Department of Clinical Laboratory, the First Affiliated Hospital of Guangxi Medical University, Nanning, Guangxi, China; 2 Department of Cardiology, the First Affiliated Hospital of Guangxi Medical University, Nanning, Guangxi, China

## Abstract

Prostate cancer is one of the most common noncutaneous malignancies in Western countries. Because there has been a debate regarding the relationship between the XRCC1-Arg399Gln and Arg280His polymorphisms and prostate cancer risk, we therefore performed this meta-analysis. The electronic databases PubMed, EMBASE, and Medline were searched prior to October 1, 2014. An odds ratio and 95% confidence interval were used to calculate association. Heterogeneity was tested by both a chi-square test and I^2^statistic. Funnel plots and Egger’s test were used to assess publication bias. All statistical analyses were performed using STATA 12.0 software. A significant association between the XRCC1-Arg399Gln polymorphism and prostate cancer risk was found under a homozygote model and a recessive model. A significant association between XRCC1-Arg280His and prostate cancer risk was found under a heterozygote model, a recessive model, and a dominant model. Overall, the results of this meta-analysis show that the XRCC1-Arg399Gln polymorphism may be associated with an increased risk for prostate cancer under the homozygote model and the recessive model. And XRCC1-Arg280His polymorphism is likely to be related with prostate cancer risk under the heterozygote model and the dominant model. Additional larger well-designed studies are needed to validate our results.

Prostate cancer (PC) is one of the most common noncutaneous malignancies in Western countries, such as Australia, the United States and France. It is considered the second most common cause of cancer death in men^1^, and its occurrence increases with age^2^,^3^. Although previous studies have reported several established risk factors such as smoking, ethnicity, UV light, inflammation, diet, age, and radiation exposure that might increase the risk of prostate cancer, there is still difficulty in definitively determining the etiology of prostate cancer^3^,^4^. Prostate cancer may not develop even after exposure to these risk factors, which suggests that genetic variations may be important contributing factors to the development of prostate cancer in addition to environmental or lifestyle factors^5^. One study showed that malignant transformation of prostate cells is always accompanied by somatic genomic changes, including deletions, amplifications, or point mutations^6^,^7^. Gene factors, particularly single nucleotide gene polymorphisms, have been reported to be closely related to the development of various malignant cancers, such as leukemia, oral cancer and prostate cancer^8^,^9^,^10^,^11^. Numerous studies have reported that single nucleotide polymorphisms in DNA repair genes may impact DNA damage and cancer risk^12^,^13^,^14^,^15^, and it is well established that DNA repair capacity may play an important role in the pathogenesis of prostate cancer^13^,^14^,^16^,^17^.

The X-ray repair cross-complementing group 1 (XRCC1) is one of the DNA repair genes. At least four DNA repair pathways operate on specific types of damaged DNA, including base excision repair (BER), nucleotide excision repair (NER), double-strand break repair and mismatched repair (MMR). BER removes small lesions, including oxidized or reduced bases, fragmented, alkylation and nonbulky adducts. Nucleotide excision repair removes larger lesions, which often result from environmental damage, including UV radiation and external carcinogens. MMR corrects replication errors (base-base or insertion-deletion mismatch) caused by DNA polymerase errors. Double-stranded DNA breaks are repaired through mechanisms involving the homologous recombination repair pathway^3^,^5^. Arg399Gln (rs25487) and Arg280His (rs25489) are two common polymorphisms of XRCC1. The functional effects of these polymorphisms in XRCC1 are not well known. The Arg399Gln polymorphism at the XRCC1 nucleotide 28152 site in exon 10 involves the amino acid substitution (glutamine to arginine). XRCC1 codon 399 is located within the BRCT domain (amino acids 301–402) that interacts with poly (ADP-ribose) polymerase (PARP). The role of XRCC1 in BER brings together DNA polymerase, DNA ligase III, and PARP at the site of DNA damage, so the 399Gln variant could have an altered repair activity^18^. In addition, the variant may be associated with several phenotypic alterations, including higher levels of sister chromatid exchange, glycophorin A mutations, polyphenol DNA adducts, aflatoxin B1–DNA adducts, and prolonged cell cycle delay^19^. The nonsynonymous Arg280His polymorphism (exon 9, base 27466 G to A, arginine to histidine) changes the amino acid sequence of XRCC1. Codon 280 of XRCC1 lies within the AP endonuclease (APE)–binding domain. This change in protein biochemistry could potentially alter the XRCC1 structure and its ability to interact with APE^20^. Takanami et al.^21^ showed that the XRCC1 (R280H) variant protein is defective in its efficient localization to a damaged site in the chromosome, which may reduce cellular BER/SSBR. These polymorphisms in DNA repair genes may also alter protein function, impair the interaction of XRCC1 with other enzymatic proteins and cause deficits in repair capacity^11^. If the damage is not repaired, it can result in genetic instability, which may contribute to unregulated cell growth and carcinogenesis. Previous studies have reported that XRCC1 polymorphisms, such as codonArg399Gln and codon Arg280His, are related to susceptibility to prostate cancer^2^,^3^,^4^,^11^,^16^,^17^,^22^,^23^,^24^,^25^,^26^,^27^,^28^,^29^. However, the conclusions have been inconsistent. Thus, in view of the uncertain association, our goal is to obtain a more precise result by conducting this meta-analysis.

## Methods

### Search strategy

We performed a comprehensive search of electronic databases, such as PUBMED, EMBASE, and Medline, using the following key words: ‘XRCC1’ OR ‘X-ray repair cross-complementing group 1,’ ‘polymorphism’ OR ‘variants,’ ‘prostate cancer or prostate carcinoma’ from their earliest available date to October 1, 2014. There were no language, sample size, time period, population, or type of report restrictions in the search. Two authors independently evaluated all associated publications to retrieve the eligible literature. In addition, the references in reviews or in eligible studies were checked to ensure that no relevant studies were missed in the database search. If a study reported a different sub-population, we treated it as a separate study in our meta-analysis^24^,^29^.

### Inclusion and exclusion criteria

Studies that met the following inclusion criteria were considered eligible articles: (1) investigated the relationship between the Arg399Gln and Arg280His polymorphisms of XRCC1 and prostate cancer risk; (2) provided sufficient data, including the genotype allele frequency in cases and controls; (3) case control studies; and (4) for multiple publications reported from the same population, we chose the largest and most complete study. Accordingly, the exclusion criteria were as follows: (1) the study was not relevant to the XRCC1 polymorphism and prostate cancer risk; (2) the study reported unavailable data; (3) the study did not offer the source of controls in the article; and (4) case reports.

### Data extraction

Two authors independently extracted the data according to the inclusion and exclusion criteria listed above. We extracted the following information from each eligible study: the name of first author, the study country, source of control, type of genotype, and sample size (cases and controls). According to the source of controls, eligible articles were defined as hospital-based or population-based. The genotype distribution was assessed according to whether it followed the Hardy-Weinberg equilibrium (HWE) balance in the control group. To guarantee the accuracy of the information extracted from the study, a third investigator reviewed the final results. Discussions were conducted to reach an agreement when we had different views on the results.

### Statistical analysis

First, all statistical analyses were performed using the STATA program (Version 12.0). For each study, the pooled odds ratio (OR) with the corresponding 95% confident interval (CI) were calculated to assess the strength of the relationship between the XRCC1-Arg399Gln polymorphism and risk of prostate cancer based on different genetic comparison models: a heterozygote model (AG vs. AA), dominant model (GG + GA vs. AA), recessive model (GG vs. AA + GA), and homozygote model (GG vs. AA). Accordingly, the relationship between theXRCC1-Arg280His polymorphism and prostate cancer risk was evaluated by the combined OR and its 95%CI based on four genetic comparison models: the heterozygote model (AH vs. AA), the dominant model (HH + HA vs. AA), the recessive model (HH vs. AA + HA), and the homozygote model (HH vs. AA). The heterogeneity among the studies was assessed by the χ^2^-test-based Q-statistic, and the degree of heterogeneity was estimated with the I^2^ statistic^30^. A significant Q-statistic (*P* < 0.10) or I^2^ > 50% indicated heterogeneity between the studies, so the pooled OR was calculated by a random-effects model. Otherwise, a fixed effect model was used. To determine the possible reasons for heterogeneity, we conducted a subgroup analysis based on ethnicity status (Africa, Caucasian or Asian) and source of control (population-based or hospital-based). The sensitivity analysis was performed by omitting each study and recalculating the pooled estimates to assess the credibility of outcomes in our meta-analysis. HWE was tested by chi-square in the control group, and a value of *P* > 0.05 showed that the controls followed HWE balance. Potential publication bias was assessed using funnel plots and Egger’s test (a *P* value less than 0.05 was considered representative of statistically significant publication bias)^31^.

## Results

### Study characteristics

**Figure 1:**
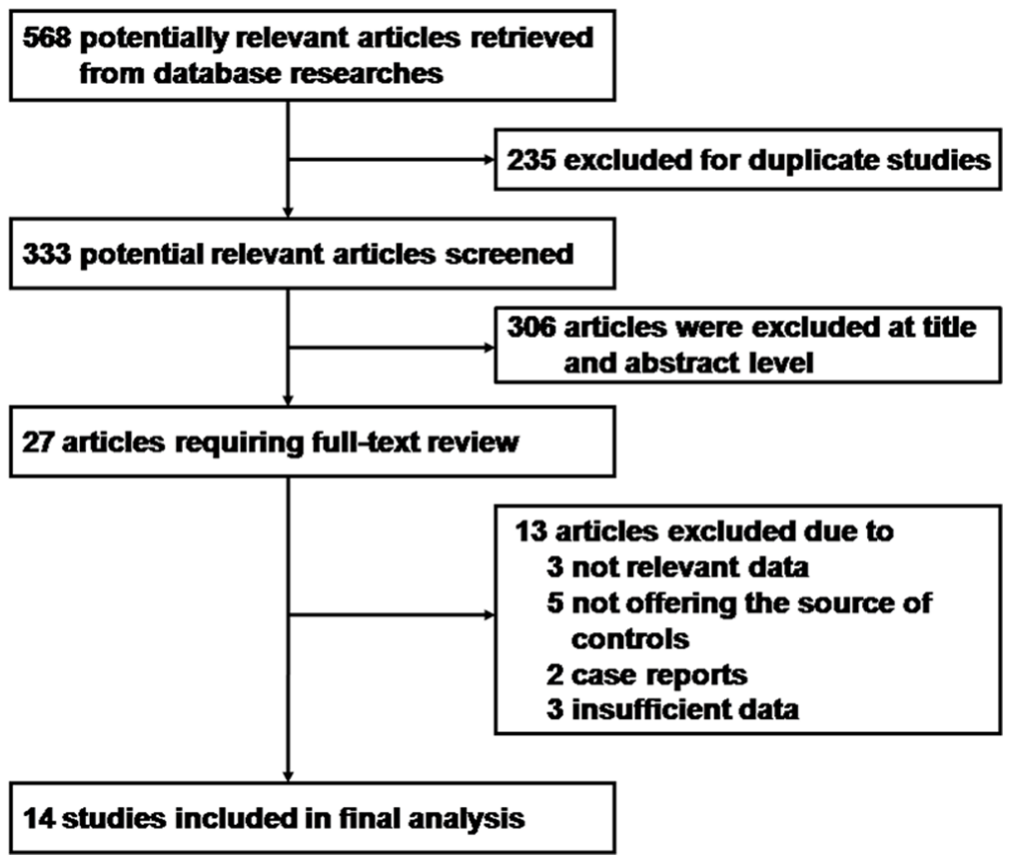
Flow chart of the study selection.

A flow chart of the detailed selection procedure is shown in Figure 1. Based on the search criteria, a total of 568 publications were located in the electronic database; 235 were excluded for duplicate studies, 306 articles were excluded after carefully screening the titles, the content and abstracts, and upon reading the full text, 27 articles were evaluated in detail. Thirteen articles were excluded because the studies had no usable data or were not relevant to the role of the XRCC1-Arg399Gln and Arg280His polymorphisms in prostate cancer risk. Finally, 14 case control publications (involving 4215 cases and 4220 controls) that met the inclusion criteria were included in our meta-analysis^2^,^3^,^4^,^7^,^11^,^16^,^17^,^23^,^24^,^25^,^26^,^27^,^28^,^29^. One study was not consistent with HWE balance^16^ (*P* < 0.05). Of the 14 case control studies, 5 were about the XRCC1-Arg280His polymorphism and susceptibility to prostate cancer; 4 were conducted in Asian populations; 7 were in Caucasian populations; and 5 were in African populations. Ten studies were population-based (PB), and the other 4 studies were hospital-based (HB). All studies were written in English. The main characteristics of the studies and the genotype distribution results of the HWE test in the control group are shown in Table 1.

### Meta-analysis results

**Figure 2:**
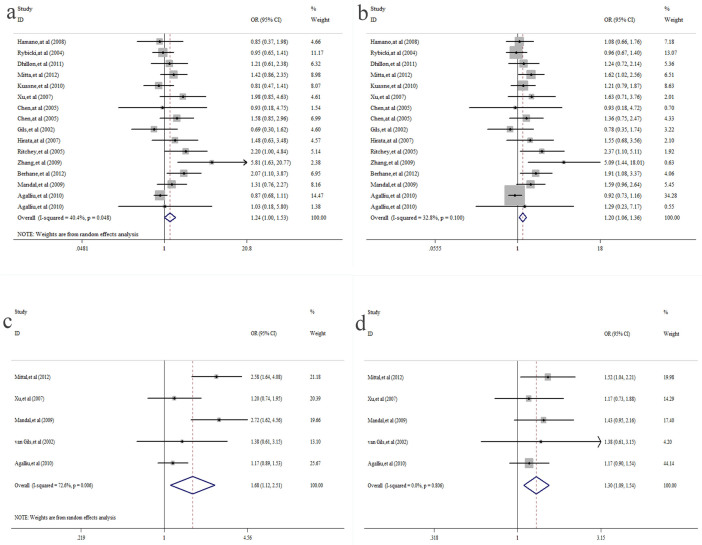
(a) Forest plot of XRCC1-Arg399Gln and prostate cancer under the homozygote model (GG vs. AA). (b) Forest plot of XRCC1-Arg399Glnand prostate cancer under the recessive model (GG vs. AA + AG). (c) Forest plot of XRCC1-Arg280His and prostate cancer under the heterozygote model (AH vs. AA). (d) Forest plot of XRCC1-Arg280His and prostate cancer under the dominant model (HH + HA vs. AA).

**Figure 3:**
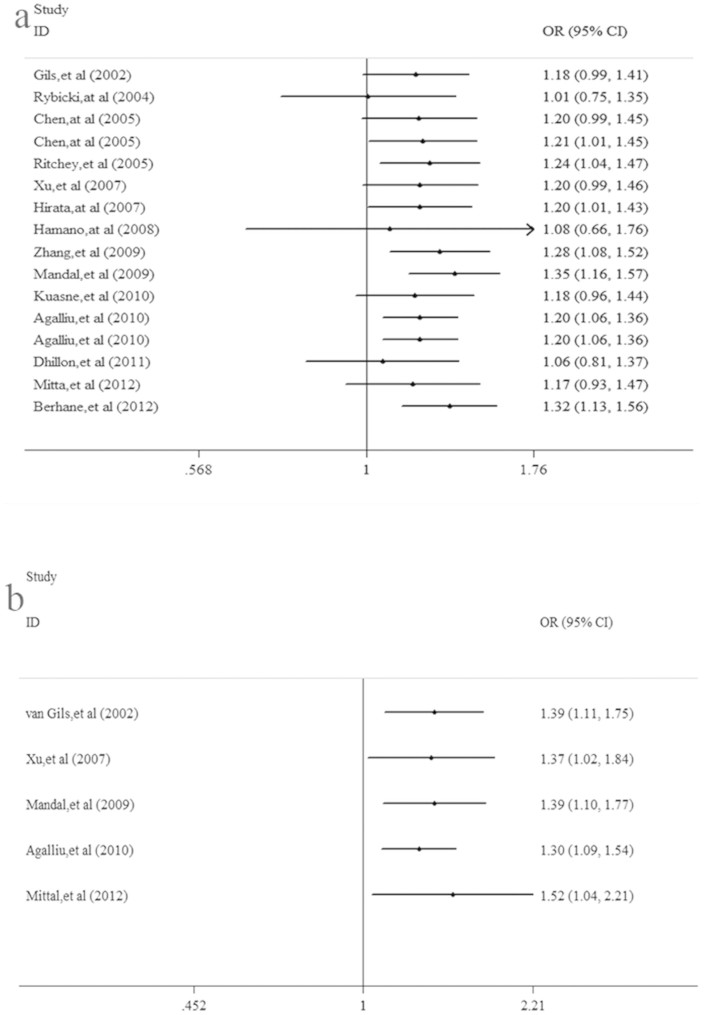
(a) Cumulative meta-analysis of the XRCC1-Arg399Gln polymorphism and prostate cancer. (b) Cumulative meta-analysis of theXRCC1-Arg280His polymorphism and prostate cancer risk.

Tables 2 and 3 summarize the main results of this meta-analysis. The pooled results based on all included studies showed a significant association between the XRCC1-Arg399Gln polymorphism and prostate cancer risk under the homozygote model GG vs. AA (OR = 1.24, 95%CI = 1.00–1.53, *P* = 0.048)(Figure 2. a) and the recessive model GG vs. AA + AG (OR = 1.20, 95%CI = 1.06–1.36, *P* = 0.005)(Figure 2. b), whereas there was no significant association under the heterozygote model AG vs. AA (OR = 0.95, 95%CI = 0.83–1.09, *P* = 0.480) or the dominant model GG + GA vs. AA (OR = 1.07, 95%CI = 0.92–1.26, *P* = 0.373). With regard to the XRCC1-Arg280His polymorphism and prostate cancer, a significant association was found under the heterozygote model AH vs. AA (OR = 1.68, 95%CI = 1.12–2.51, *P* = 0.012)(Figure 2. c), the recessive model HH vs. AA + HA (OR = 0.58, 95%CI = 0.42–0.80, *P* = 0.001), and the dominant model HH + HA vs. AA (OR = 1.30, 95%CI = 1.09–1.54, *P* = 0.004)(Figure 2. d). However, there was no significant difference under the homozygote model HH vs. AA (OR = 0.81, 95%CI = 0.57–1.13, *P* = 0.213). Furthermore, in the subgroup analysis by ethnicity, we discovered that there was no significant association in Caucasian populations in any of the gene models (*P* > 0.05). A significant association between the XRCC1-Arg399Gln polymorphism and prostate cancer was observed in African and Asian populations under only the homozygote model and the recessive model. When stratifying the studies by source of control, we found an increased risk in the PB group under the recessive model GG vs. AA + AG (OR = 1.22, 95%CI = 1.05–1.43, *P* = 0.012). There was no significant association in the HB group in any of the gene models (*P* > 0.05). The detailed results are shown in Table 2. We also performed a cumulative meta-analysis based on publication time, which further confirmed our results (Figure 3. a&b).

### Sensitivity analysis

**Figure 4:**
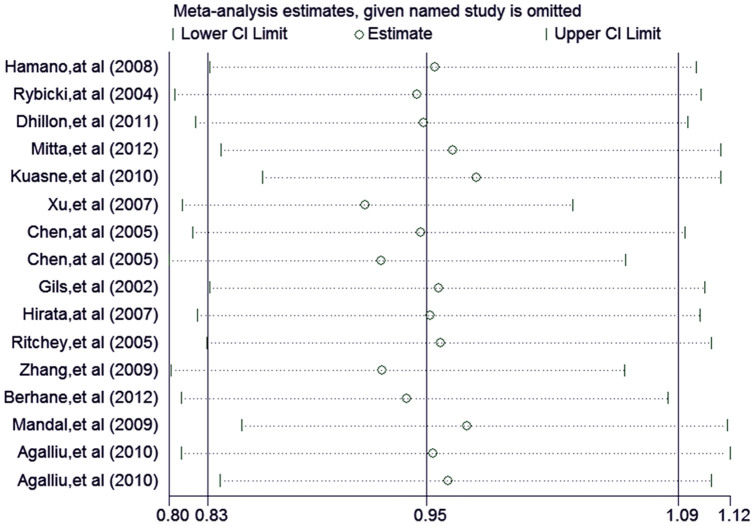
Sensitivity analysis of the XRCC1-Arg399Gln polymorphism and prostate cancer risk.

A sensitivity analysis was conducted to assess the stability of the overall results through the sequential omission of each individual study. In our meta-analysis, the results show that no single study could affect the overall results, which indicated the reliability of our results (Figure 4).

### Publication bias

**Figure 5:**
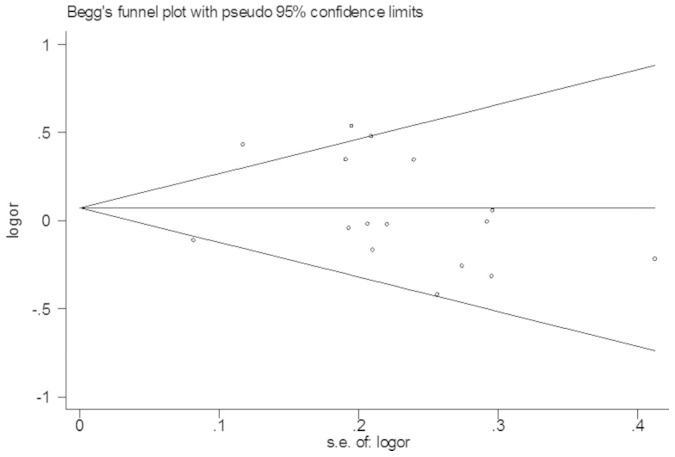
Funnel plot for publication bias in the meta-analysis of the XRCC1-Arg399Gln polymorphism and prostate cancer risk.

The funnel plot and Egger’s test were conducted to assess the potential publication bias of the studies. The shapes of the funnel plots did not show any evidence of obvious asymmetry (Figure 5). Similarly, Egger’s test was used to provide statistical evidence of funnel plot symmetry. The *P* values from Egger’s test were greater than 0.05, suggesting no publication bias in our meta-analysis.

## Discussion

A variety of exogenous and endogenous factors can constantly damage cellular DNA. Unrepaired damage can result in the formation of DNA lesions or apoptosis or may even lead to unregulated cell growth and cancer. Several responses may occur to prevent replication in the presence of genetic errors in vivo^32^. DNA repair genes play a critical role in maintaining genome integrity and repairing this damage. Currently, there is increasing evidence that gene analysis reveals the importance of DNA repair genes and that polymorphisms, such as the XRCC1 polymorphisms R399Q and R280H, are associated with different human cancers, such as bladder cancer, breast cancer, colorectal cancer, lung cancer, and endometrial carcinoma^16^,^33^,^34^,^35^,^36^. These polymorphisms have also been explored in numerous studies for an association with prostate cancer, with conflicting results. We think that there are most likely several limitations to these studies, such as different study population ethnicities or small sample sizes. A meta-analysis is considered a powerful method for summarizing controversial results from different studies, thereby affording more statistical power by increasing the number and size of datasets, which can reduce the probability of random error and generating false positive or false negative associations, obtaining more precise and reliable results than an individual study^37^.

This meta-analysis addressed the association between two polymorphisms in the XRCC1 gene and prostate cancer risk, involving 2124 cases and 2219 controls concerning the Arg280His polymorphism from 5 studies, a pooled total of 4215 patients with prostate cancer, and 4220 controls concerning the Arg399Gln polymorphism from 14 studies. The overall data showed that the XRCC1 Arg399Gln polymorphism may be a risk factor for prostate cancer under the homozygote model (GG vs. AA) and the recessive model (GG vs. AA + AG), indicating that individuals who have the Gln allele are more likely to have prostate cancer (homozygote model: OR = 1.24, 95%CI:1.00–1.53; recessive model: OR = 1.20, 95% CI:1.06–1.36). In the subgroup analysis by ethnicity, we discovered that Arg399Gln had a significant correlation with prostate cancer in Asian (homozygote model: OR = 1.55, 95%CI: 1.02–2.35; recessive model: OR = 1.43, 95% CI: 1.02–2.00) and African populations (homozygote model: OR = 1.48, 95%CI: 1.09–2.01; recessive model: OR = 1.64, 95% CI: 1.23–2.18). A previous meta-analysis performed by Geng et al.^38^ showed a significant association between the XRCC1 Arg399Gln polymorphism and prostate cancer risk in Asian subjects under a recessive genetic model in a stratified analysis by ethnicity. Another study conducted by Wei et al.^39^ found that the XRCC1 399Gln allele might be a low-risk factor for prostate cancer only in Asian men under a homozygote model and a recessive model. For the Arg280His polymorphism and prostate cancer risk, there was a significant association under the heterozygote model and the dominant model in all populations.

Many studies have extensively investigated the XRCC1 Arg399Gln polymorphism and have reported that this polymorphism is related to different cancers in Asian populations, including gastric cancer^40^, bladder cancer^41^, lung cancer^42^, and colorectal cancer^43^. Accordingly, there have been a wide variety of studies regarding the XRCC1 Arg399Gln polymorphism in Africa involving breast cancer^44^, and colorectal cancer^43^ among others. However, Wang et al.^45^ reported that no association was found between the XRCC1-Arg399Gln polymorphism and risk of colorectal cancer. Genetic polymorphism often varies with ethnicity. Some genetic polymorphisms may be associated with a risk of some types of cancers in a certain ethnicity; it has been reported that XRCC1 Arg399Gln is associated with lung cancer among Asians but not among individuals from Western countries^46^,^47^. Furthermore, Zeng et al.^48^ suggested that a statistically significant association between the XRCC1 Arg399Gln polymorphism and colorectal cancer risk was observed in Asians but not in Caucasians. A previous study showed that 399Glu/Glu may increase breast cancer risk in African populations but not in Caucasians^49^,^50^. These different results may result from the possible role of ethnic differences in genetic backgrounds, and additionally, other factors such as different types of diets or lifestyles or the interaction between genes and the environment may also play a role.

There have been studies regarding the XRCC1 Arg280His polymorphism and other cancers in different populations. These studies have suggested that the XRCC1 280His polymorphism is associated with hepatocellular carcinoma (HCC) risk and bladder cancer in Asian populations^51^,^52^. An increased risk of advanced colorectal neoplasia in individuals with the XRCC1 Arg280His polymorphism in white populations has also been reported^53^. In African populations, the Arg280His polymorphism of XRCC1 has been associated with breast cancer^54^, though such results for XRCC1 Arg280His are still considered controversial. Arg280His has also been shown to not be a risk factor for breast cancer and bladder cancer^55^,^56^, and no association has been found between lung cancer risk and the XRCC1 Arg280His polymorphism^57^. However, one study did show that the XRCC1 Arg280His allele was associated with increased lung cancer risk after adjustment for radon and tobacco exposure^58^. That result might be a reflection of gene-environment interactions or a pattern of linkage disequilibrium, which is common with other more important polymorphisms. We included five studies describing the association between the Arg280His polymorphism and prostate cancer and did not conduct a subgroup analysis by ethnicity. We would need a larger sample size and different populations to assess gene-environment studies and obtain more comprehensive and accurate results.

Considerable heterogeneity was detected among the studies. We also performed a subgroup analysis according to the source of the control, but the heterogeneity could not be fully explained. The included studies might have sample selection bias, and the different characteristics of subjects and their ethnic backgrounds may have a certain influence. In addition, hospital-based controls might not be truly representative of the general population^59^, which may affect the results. A strict matching criteria is needed for a proper control, which is important for reducing possible selection bias.

Although we performed a comprehensive analysis, several potential limitations should be considered. First, the genotype distributions in the control group from one study did not follow HWE balance^16^, so the results may be biased. Second, heterogeneity among the studies may disturb the results of the meta-analysis. A third limitation is that the people in the control groups were primarily healthy subjects, but some specific genetic characteristics may have had an effect among these controls, and we could not entirely rule out the possibility of the risk of prostate cancer. Finally, 5 studies were too small to evaluate a role of the Arg280His polymorphism in prostate cancer risk. The differences observed in this meta-analysis may be caused by chance with such small sample sizes. Thus, the results regarding the relationship between the Arg280His polymorphism and prostate cancer should be treated cautiously.

In summary, our meta-analysis evaluated all of the available published data, and the results suggested that a significant association between XRCC1-Arg399Gln and risk of prostate cancer was found under the homozygote model and the recessive model, particularly in Asian and Africa populations. A significant association between the XRCC1-Arg280His polymorphism and prostate cancer susceptibility was found under the heterozygote model and the dominant model. In view of some limitations of our meta-analysis, well-designed case control studies and larger population sizes are needed to validate the role of XRCC1 Arg399Gln and Arg280His polymorphisms in the development of prostate cancer.

## Author Contributions

F.L., H.T. and Z.D. defined the research theme. J.Y., X.W. and W.Y. designed the methods, analyzed the data, prepared figures and tables. J.Y. wrote the main manuscript text. All authors reviewed the manuscript.

## Figures and Tables

**Table 1:** Characteristics of studies included in the Meta-analysis

ID	First author	Country	Type of genotype	Source of control	Total		HWE(No/Yes)
					Case	Control	
1	Hamano	Japan	XRCC1-Arg399Gln	PB	142	119	Y
2	Rybicki	America	XRCC1-Arg399Gln	HB	637	480	Y
3	Dhillon	Australian	XRCC1-Arg399Gln	HB	118	132	Y
4	Mittal	India	XRCC1-Arg399Gln XRCC1-Arg280His	PB	212	250	N
5	Kuasne	Brazil	XRCC1-Arg399Gln	HB	172	172	Y
6	Xu	China	XRCC1-Arg399Gln XRCC1-Arg280His	PB	207	235	Y
7	Chen	America	XRCC1-Arg399Gln	HB	352	335	Y
8	Gils	Italy	XRCC1-Arg399Gln XRCC1-Arg280His	PB	77	183	Y
9	Hirata	Japan	XRCC1-Arg399Gln	HB	165	165	Y
10	Ritchey	China	XRCC1-Arg399Gln	PB	162	251	Y
11	Zhang	America	XRCC1-Arg399Gln	PB	193	197	Y
12	Berhane	India	XRCC1-Arg399Gln	PB	150	150	Y
13	Mandal	India	XRCC1-Arg399Gln XRCC1-Arg280His	PB	171	200	Y
14	Agalliu	America	XRCC1-Arg399Gln XRCC1-Arg280His	PB	1457	1351	Y

PB: population-based; HB: hospital-based; HWE: Hardy-Weinberg equilibrium.

**Table 2:** Meta-analysis of the association between XRCC1-Arg 399 Gln polymorphism and prostatic cancer risk

	AG vs. AA				GG vs. AA				Recessive model				Dominant model				
	OR(95%CI)	P	P_het_	I^2^(%)	OR(95%CI)	P	P_het_	I^2^(%)	OR(95%CI)	P	P_het_	I^2^(%)	OR(95%CI)	P	P_het_	I^2^(%)	
**Overall**	0.95(0.83,1.09)	0.480	0.043	41.3	1.24(1.00,1.53)	0.047	0.048	40.4	1.20(1.06,1.36)	0.005	0.100	32.8	1.07(0.92,1.26)	0.373	0.001	59.2	
**Ethnicity**																	
Asian	1.03(0.70,1.52)	0.873	0.061	59.3	1.55(1.02,2.35)	0.039	0.388	0.8	1.43(1.02,2.00)	0.038	0.383	1.9	1.13(0.81,1.58)	0.468	0.110	50.3	
Caucasian	0.98(0.80,1.19)	0.817	0.062	49.9	1.06(0.79,1.42)	0.720	0.058	50.7	1.05(0.89,1.23)	0.567	0.146	37.1	1.10(0.85,1.44)	0.466	0.000	76.4	
Africa	0.82(0.65,1.03)	0.086	0.480	0.0	1.48(1.09,2.01)	0.012	0.779	0.0	1.64(1.23,2.18)	0.001	0.934	0.0	0.97(0.79,1.19)	0.774	0.433	0.0	
**Source of control**																	
PB	0.95(0.78,1.15)	0.576	0.040	49.0	1.38(0.99,1.91)	0.056	0.011	58.1	1.22(1.05,1.43)	0.012	0.016	55.7	1.05(0.87,1.26)	0.647	0.019	54.5	
HB	0.95(0.76,1.20)	0.692	0.165	36.2	1.07(0.84,1.37)	0.585	0.622	0.0	1.16(0.93,1.44)	0.196	0.869	0.0	1.12(0.86,1.46)	0.409	0.035	58.4	

PB: population-based; HB: hospital-based.

**Table 3:** Meta-analysis of the association between XRCC1-Arg280His and prostate cancer risk

Genetic model	Test of association		Test of heterogeneity	
	OR (95%CI)	P-value	P-value	I^2^(%)
AH vs. AA	1.68(1.12,2.51)	0.012	0.006	72.6
HH vs. AA	0.81(0.57,1.13)	0.213	0.901	0.0
Recessive model	0.58(0.42,0.80)	0.001	0.735	0.0
Dominant model	1.30(1.09,1.54)	0.004	0.806	0.0
